# Comparison of Global Gene Expression of Gastric Cardia and Noncardia Cancers from a High-Risk Population in China

**DOI:** 10.1371/journal.pone.0063826

**Published:** 2013-05-22

**Authors:** Gangshi Wang, Nan Hu, Howard H. Yang, Lemin Wang, Hua Su, Chaoyu Wang, Robert Clifford, Erica M. Dawsey, Jian-Min Li, Ti Ding, Xiao-You Han, Carol Giffen, Alisa M. Goldstein, Philip R. Taylor, Maxwell P. Lee

**Affiliations:** 1 Genetic Epidemiology Branch, Division of Cancer Epidemiology and Genetics, NCI, Bethesda, Maryland, United States of America; 2 Office of the Director, Center for Cancer Research, NCI, Bethesda, Maryland, United States of America; 3 Multidrug Resistant Organism Repository and Surveillance Network, Walter Reed Army Institute of Research, Silver Spring, Maryland, United States of America; 4 Shanxi Cancer Hospital, Taiyuan, Shanxi, PR China; 5 Information Management Services, Inc., Silver Spring, Maryland, United States of America; Duke-National University of Singapore Graduate Medical School, Singapore

## Abstract

**Objective:**

To profile RNA expression in gastric cancer by anatomic subsites as an initial step in identifying molecular subtypes and providing targets for early detection and therapy.

**Methods:**

We performed transcriptome analysis using the Affymetrix GeneChip U133A in gastric cardia adenocarcinomas (n = 62) and gastric noncardia adenocarcinomas (n = 72) and their matched normal tissues from patients in Shanxi Province, and validated selected dysregulated genes with additional RNA studies. Expression of dysregulated genes was also related to survival of cases.

**Results:**

Principal Component Analysis showed that samples clustered by tumor vs. normal, anatomic location, and histopathologic features. Paired t-tests of tumor/normal tissues identified 511 genes whose expression was dysregulated (*P*<4.7E-07 and at least two-fold difference in magnitude) in cardia or noncardia gastric cancers, including nearly one-half (n = 239, 47%) dysregulated in both cardia and noncardia, one-fourth dysregulated in cardia only (n = 128, 25%), and about one-fourth in noncardia only (n = 144, 28%). Additional RNA studies confirmed profiling results. Expression was associated with case survival for 20 genes in cardia and 36 genes in noncardia gastric cancers.

**Conclusions:**

The dysregulated genes identified here represent a comprehensive starting point for future efforts to understand etiologic heterogeneity, develop diagnostic biomarkers for early detection, and test molecularly-targeted therapies for gastric cancer.

## Introduction

Gastric cancer is the fourth most common cancer and the second most frequent cause of cancer death worldwide [1]. As a result of its large population and high rates, China accounts for 42% of all gastric cancer deaths in the world each year [1]. Shanxi Province is one of the regions with the highest incidence rate of gastric cancer in China [2], [3]. In fact, gastric cancer remains the leading cause of death from cancer in both men (36%) and women (28%) in this region [4], despite the decline in incidence for this cancer in northern China.

Gastric cancer rates in China are highest in the north and risk factors for both cardia and noncardia gastric cancers have been previously studied there. Increased age, male gender, a family history of upper gastrointestinal tract cancer, tobacco exposure, and *Helicobacter pylori* infection have all been consistent risk factors for both gastric cardia adenocarcinoma (GCA) and gastric noncardia adenocardinoma (GNCA) [5]–[13]; additionally, emerging evidence supports increased risk from thermal damage from hot food [6]. Diet, particularly micronutrients, appear to play an important protective role, as evidenced by results from a large, randomized controlled trial conducted in Linxian which showed reduced GCA and GNCA mortality from the antioxidant combination of selenium, vitamin E, and beta-carotene [14], [15]; other questionnaire-based nutritional studies also support the role of nutrition in gastric cancer etiology [6].

Gastric cancers are histopathologically classified into diffuse and intestinal types [16] for both cardia and noncardia. Anatomically, the cardia lies between the end of the esophagus and the body of the stomach, and is a small macroscopically indistinct zone immediately distal to the gastro-esophageal junction. It merges distally into the fundus and is distinguishable only by its histological pattern.

In addition to being anatomically adjacent, GCA and esophageal squamous cell carcinoma (ESCC) both occur at epidemic rates in this population, share some etiologic risk factors, and before the widespread use of endoscopy and biopsy, were diagnosed as a single disease referred to as “esophageal cancer” or “hard swallowing disease” [17]. The ability to diagnose GCA and accurately distinguish it from ESCC has led to an increase in the incidence in gastric cancer in this region [18]. The reason for the high rates of GCA and ESCC in this geographic area and their relation to each other remains unclear, but there are almost certainly common etiologically important environmental exposures, and a recent genome-wide association study of germline DNA found a common gene (*PLCE1*) associated with risk for both GCA and ESCC [19].

Gastric adenocarcinomas are a heterogeneous group of tumors. GCAs show biologic, epidemiologic, and clinicopathologic features that more closely resemble esophageal adenocarcinomas (EACs) than GNCAs, suggesting that tumors arising in the stomach may have distinct etiologies. For example, several studies detected substantially higher *TP53* mutation rates in cases with GCA than GNCA, while the *TP53* mutation spectrum in GCA more closely resembled EAC [20]. A number of other genetic alterations have been reported in gastric cancer, including *CDH1* [21], *β-catenin* [22], *TFF1* [23], and *Met* [24], but no study compared these alterations by anatomic subsite. Further, although several gastric cancer gene expression profiling studies have previously been reported [25]–[31], none has directly compared GCA and GNCA cases from a high-risk geographic region using a common protocol.

The objective of this study was to identify genomic differences between gastric cancer by anatomic subtypes to aid our understanding of the etiologies of these two distinct cancers and facilitate the development of appropriate targeted strategies for early detection, prognosis, and therapy.

## Materials and Methods

### 1. Patient Selection and Follow-up

This study was approved by the Institutional Review Boards of the Shanxi Cancer Hospital in China and the National Cancer Institute (NCI) in the USA. Patients admitted to the Shanxi Cancer Hospital between 1998 and 2001 with a diagnosis of GCA or GNCA and considered candidates for curative surgical resection were identified and recruited to participate in the study. None of the patients had prior therapy, and Shanxi was the ancestral home for all. After obtaining informed consent, patients were interviewed to obtain information on demographic and lifestyle cancer risk factors and clinical data and samples were obtained.

Gastric cancer here was defined by histology (only adenocarcinomas were included) and anatomic sites were defined as gastric cardia adenocarcinoma (GCA) and gastric noncardia adenocarcinoma (GNCA). Cardia cancers were gastric cancers located in the proximal three centimeters of the stomach (C16.0), while noncardia cancers were those in the remainder of the stomach (fundus, body, antrum, pylorus (C16.1-8), and unspecified location (C16.9) based on site codes from the International Classification of Malignant Tumours (Seventh edition)) [32].

Between 2005 and 2007, all patients (or their families) from this study were re-contacted to ascertain vital status. For those who had died, date and cause of death were determined.

### 2. Sample Collection

Tumor and matched normal tissues obtained during surgery were snap-frozen in liquid nitrogen and stored at −130 degrees C until used. Cases were chosen for this study based on three criteria: (i) histological diagnosis of GCA or GNCA confirmed by pathologists at both the Shanxi Cancer Hospital and the NCI; (ii) tumor samples that were at least 50% tumor; and (iii) tissue RNA quality/quantity adequate for testing.

### 3. Sample Preparation and Chip Hybridization

Total RNA was extracted from frozen tumor and matched normal tissues using TRIzol reagent (Invitrogen, Carlsbad, CA) in accordance with the manufacturer’s instructions. RNA purification was performed according to the manufacturer’s instructions for the RNeasy Mini Kit (Qiagen Inc, Valencia, CA) and RNase-Free DNase Set digestion (Qiagen Inc, Valencia, CA). RNA quality and quantity were determined using the RNA 6000 Labchip/Agilent 2100 Bioanalyzer (Agilent Technologies, Germantown, MD).

Microarray experiments were performed using 8 µg total RNA; details of reverse transcription, labeling, and hybridization were according to the manufacturer’s protocol (http://www.affymetrix.com/support/technical/manual/expression_manual.affx; accessed 2013 Apr 14). Briefly, the procedures included first strand cDNA synthesis, second strand cDNA synthesis, double-stranded cDNA clean up, *in vitro* transcription, cRNA purification, and fragmentation. Twenty µg biotinylated cRNA were used in each array hybridization. Samples were hybridized onto Affymetrix GeneChip Human Genome U133A chips (Affymetrix, Santa Clara, CA). After hybridization at 45°C overnight, arrays were subsequently developed with phycoerythrin-conjugated streptavidin by fluidics station (GeneChip Fluidics Station 450, Santa Clara, CA) and were scanned (GeneChip Scanner 3000, Santa Clara, CA) to obtain quantitative gene expression levels. Paired tumor and normal tissue specimens from each patient were processed simultaneously throughout the experimental process. The average present call for the 124 chips from the 62 GCA patients was 50.0%; for the 144 chips from the 72 GNCA patients it was 51.5%.

### 4. Statistical Analysis

There are 22,283 probe sets on the Affymetrix GeneChip Human Genome U133A (HG_U133A). The Robust Multiarray Average (RMA) algorithm [33], [34] implemented in Bioconductor in R (http://www.bioconductor.org; accessed 2013 Apr 14) was used for background correction and normalization across all samples. All statistical methods were developed in R. The GEO accession number for these array data is GSE29272.

Principal Components Analysis (PCA) was used for clustering analysis of all gastric cancer samples analyzed here. For the PCA only, we also included data from a recently published expression array study of ESCC cases for comparison [35].

We applied paired t-tests to each of the 22,283 probesets to identify genes that were differentially expressed between tumors and their matched normal samples, but we present results only for the 21,130 probesets that mapped to 13,003 genes. To account for multiple comparisons, we selected genes that showed significant differences with *P*-values less than 4.73E-07 (equal to 0.01 divided by 21,130, ie, a conservative Bonferroni adjustment). In addition to the *P*-value cutoff, differentially-expressed genes had to show at least a two-fold difference in gene expression magnitude between tumor and normal tissues (ie, fold change either ≥2 or ≤0.50).

To identify dysregulated genes whose expression was associated with personal (gender and family history of upper gastrointestinal or UGI cancer) and clinical (tumor stage, grade, lymph node metastasis) characteristics, we performed unpaired t-tests for gene expression differences between samples using the ratio of tumor gene expression divided by the matched normal gene expression. A *P*-value threshold (*P*<0.005) was used for significance for these analyses; no fold change criteria were applied.

To assess the relation of gene expression to survival, Kaplan-Meier (KM) plots were used to visualize survival differences by high (above median) vs low (below median) gene expression status and log-rank tests were used to test for differences using the tumor probeset signal for each differentially-expressed gene identified in the tumor/normal paired t-test analysis described above. Genes whose expression was significantly related to survival in log-rank tests were further evaluated in Cox proportional hazard models for high vs low expression with adjustment for demographic and clinical characteristics of tumors (ie, age, sex, stage, grade, metastasis). For all survival analyses, we used a two-sided *P*-value <0.05 as our threshold for statistical significance.

### 5. Validation of Differentially-Expressed Genes Using Quantitative Real-Time RT-PCR

A total of 21 differentially-expressed genes (12 for GCA and 9 for GNCA) were selected for validation using quantitative Real-Time RT-PCR (qRT-PCR). For technical validation, qRT-PCR assays were performed using the same tumor and normal RNAs analyzed in the microarray experiment for a subset of GCAs (n = 41 of 62) and GNCAs (n = 50 of 72). For replication validation, tumor and matched normal RNAs from a new set of GNCAs (n = 44) were tested.

First strand cDNA was synthesized using 3 µg total RNA with Oligo (dT)_12−18_ (500 µg/ml) in a 20 µl reaction with Superscript II reverse transcriptase system (Invitrogen, Carlsbad, CA). The cDNA products were then diluted at 1∶100. Real-time PCR reactions were performed using an ABI Prism 7000 Sequence Detection System (Perkin-Elmer Applied Biosystems, Foster City, CA). All primers and probes of seven target genes and an internal control gene glyceraldehyde-3-phosphate dehydrogenase (*GAPDH*) were purchased from Applied Biosystems. qRT-PCR reactions were carried out according to the manufacturer’s protocol, as described previously [36]. The thermal cycling conditions included an initial denaturing step at 95°C for 10 min, 40 cycles at 95°C each for 15 sec, 60°C for one min, and 72°C for one min. Gene expression was analyzed using 2^–ΔΔCT^ algorithm.

## Results

### 1. Patient Characteristics

A total of 62 GCA and 72 GNCA patients were analyzed using the Affymetrix U133A array. Personal and clinical data for patients studied are summarized in **Table 1** (Clinical characteristics for individual cases studied here are shown in **Table S1**). The average age at diagnosis was mid-to-late 50s, males predominated, and about one-fourth had a family history of upper gastrointestinal (UGI) cancer (ie, esophageal or gastric cancer in first, second, or third degree relatives). For both GCA and GNCA, most cases studied were late stage, high grade, and intestinal cell type tumors with lymph node metastasis. Median survival for GCA cases was 20.3 months, and for GNCA cases was 27.8 months, based on a total of 50 and 52 deaths, respectively.

**Table 1:** Summary of personal and clinical characteristics of gastric cardia adenocarcinoma (GCA) and gastric noncardia adenocarcinoma (GNCA) cases.

Characteristic	GCA cases(n = 62)		GNCA cases (n = 72)	
Age (average years)		58.8	55.0	
Gender (fraction male)		0.73	0.81	
Family history of UGI cancer (fraction positive)		0.26	0.22	
Tumor stage (fraction)				
I		0.02	0.04	
II		0.00	0.08	
III		0.90	0.81	
IV		0.08	0.07	
Tumor grade (fraction)				
1		0.02	0.00	
2		0.42	0.29	
3		0.56	0.71	
Lymph node metastasis (fraction positive)		0.81	0.75	
Dead (fraction deceased)		0.80	0.72	
Survival (median, months)		20.3	27.8	

### 2. Principal Component Analysis of Gene Expression Microarray Data

**Figure 1:**
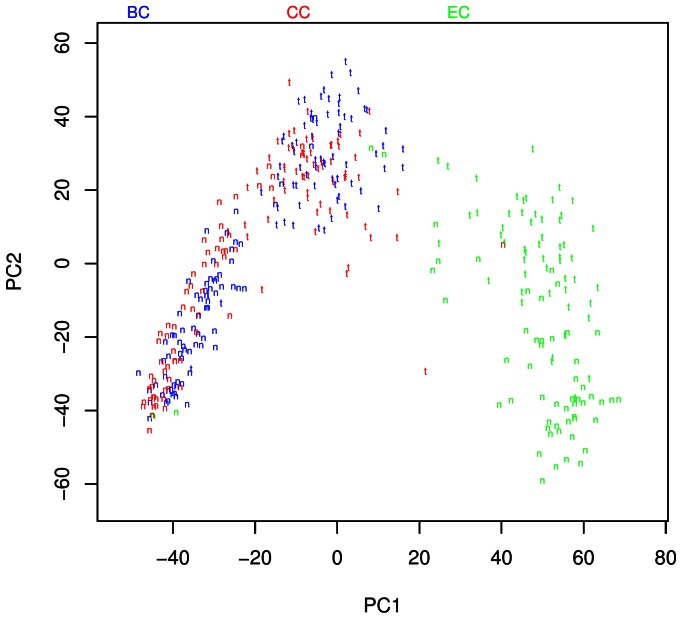
Principle component analyses of RNA expression for gastric cardia, gastric noncardia, and esophageal cancers. PCA revealed two major clusters of samples separating gastric cancer (CC [GCA] in red, N = 62; BC [GNCA] in blue, N = 72) from EC [ESCC] (green, N = 53) in the PC1 axis. PC2 further divided clusters into tumor (t) and normal (n) samples. (Note regarding comparability of expression results: Cases of ESCC, GCA, and GNCA were enrolled concurrently from a single hospital using a common protocol; samples were collected, processed, stored, and transported in an identical manner; and laboratory analyses were performed during the same time period, in the same lab, by the same technical staff, using the same platform, and the same technical approach.).

**Figure 2:**
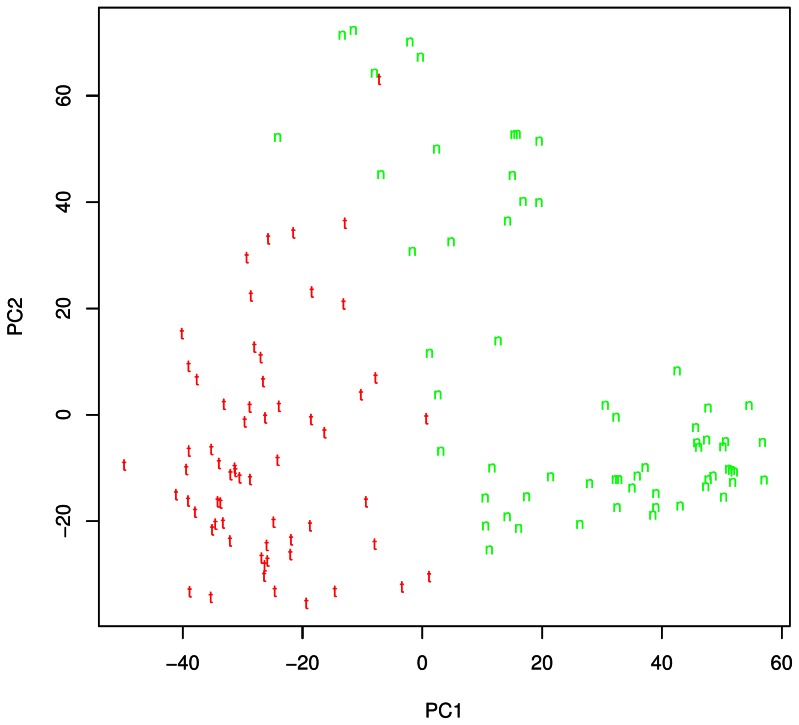
PCA analysis of GCA patients (124 chips from 62 patients). Red “t “ represents tumor and green “n” represents matched normal tissue.

**Figure 3:**
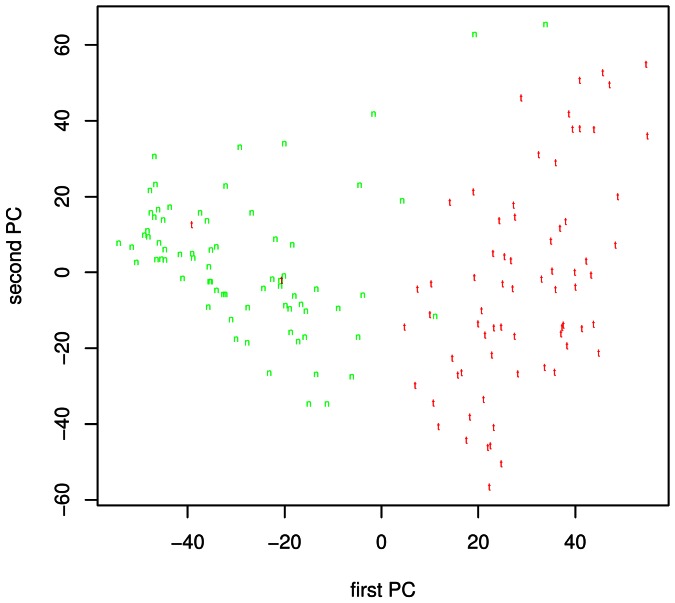
PCA analysis of GNCA patients (144 chips from 72 patients). Red “t “ represents tumor and green “n” represents matched normal tissue.

We used Principal Component Analysis (PCA) to gain an understanding of global gene expression of these samples. In this analysis, we evaluated 124 samples from 62 GCA patients (each with tumor and normal pair) and 144 samples from 72 GNCA patients (each with tumor and normal pair) as well as 106 samples from ESCC patients (each with tumor and normal pair) from a previous study [35]. PCA revealed two major clusters of samples separating all gastric cancers combined (GCA in red, GNCA in blue) from ESCC (green) in the PC1 axis **(** **Figure 1** **)**. The two clusters were further divided into normal and tumor tissues by PC2 (tumor and normal are denoted by t and n, respectively). The difference between GCA (red) and GNCA (blue) was also noticeable, especially for normal tissues. We then concentrated on the analyses of gastric cancer. PCA of GCA **(** **Figure 2** **)** and GNCA **(** **Figure 3** **)** showed again the separation of samples into tumor (t) and normal (n) clusters.

### 3. Identification of Genes Up- or Down-Regulated in GCA and GNCA

**Figure 4:**
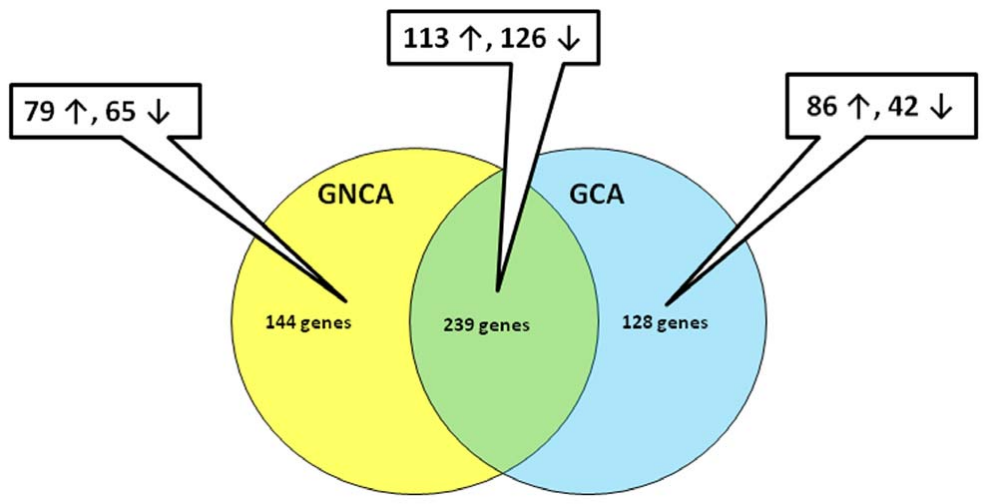
Diagram showing the number of significantly dysregulated genes (GCA only, GNCA only, and common to both GCA and GNCA).

For GCA, a total of 367 genes were differentially expressed between tumors and their matched normal samples. Of these genes, 199 genes were up-regulated and 168 were down-regulated **(** **Figure 4** **)**. For GNCA, a total of 383 genes were differentially expressed between tumors and matched non-tumor samples, including 192 genes up-regulated and 191 genes down- regulated **(** **Figure 4** **)**.

### 4. Comparison of Gene Expression in GCA and GNCA

We compared the two sets of genes that showed significant differences in gene expression for GCA and GNCA and identified 239 genes that were dysregulated in both GCA and GNCA, among which 113 were up- and 126 down-regulated **(** **Figure 4** **and Table S2)**. In addition, we found that 128 genes were dysregulated only in GCA (86 up- and 42 down-regulated) **(** **Figure 4** **and** **Table S3)**, and 144 genes were dysregulated only in GNCA (79 up- and 65 down-regulated) **(** **Figure 4** **and Table S4)**.

Among the 113 genes up-regulated in both GCA and GNCA were genes associated with cell cycle checkpoint (eg, *CDC2, TOP2A*), Wnt signaling (eg, *SULF1*, *SFRP4, LEF1, LAMB1*), adhesion (eg, *FN1*), and the TGF-β pathway (eg, *COL1A1*, *COL1A2*, *COL3A1*). In contrast, the 126 down-regulated genes common to both GCA and GNCA were enriched in processes involved in metabolism (eg, *AADAC*, *CA2*, *CA9*), digestion (eg, *ATP4A, ATP4B*), and the development of gastrointestinal tissue (eg, *GIF*, *MUC5A*, *MUC6*, *TFF1*, *TFF2*) **(Table S2)**. These results suggest that GCA and GNCA share many common etiologic pathways.

Genes up-regulated only in GCA were involved in cell cycle checkpoint regulation (eg, *CCNB1*, *CCNB2*, *CCNE1, CDC25B, SFN*), CXC chemokine (eg, *CXCL1*, *CXCL10*), and extracellular matrix (eg, *MMP9, MMP11*); while GCA-only down-regulated genes were associated with detoxification (eg, *GPX3*) and oncogenes (eg, *FOS*, *JUN*) **(Table S3)**.

Among genes altered significantly in GNCA only, up-regulated genes included those related to epithelial-to-mesenchymal transition (eg, *CALD1*, *IGFBP4*) and actin filaments (eg, *DES, FLNA*). By contrast, down-regulated genes functioned in detoxification (eg, *CYP2C9*) and epithelial surfaces (eg, *MUC1*) **(Table S4)**.

### 5. Relation Between Gene Expression and Patient Personal/Clinical Characteristics

In GCA, differentially-expressed genes were found to be related to family history of UGI cancer **(Table S5a)** and lymph node metastasis **(Table S5b)**, but not other characteristics (ie, gender, tumor stage, tumor grade; data not shown). Sixty-seven genes were significantly dysregulated (47 up- and 20 down-regulated) in patients with a family history of UGI cancer (n = 16 cases) compared to patients without such history (n = 46 cases), but fold changes were generally small: the largest fold change among up-regulated genes was 1.39 (ie, *JDP1*), while four down-regulated genes (*LMO4*, *ABHD2, LAMA3*, *MAP17*) were reduced by one-third or more **(Table S5a).** For clinical characteristics, we identified 57 genes that were significantly dysregulated (9 up- and 48 down-regulated) in GCA patients with positive lymph nodes (n = 50 cases) compared to lymph node negative patients (n = 12 cases); 11 of these genes (six down- and five up-regulated) reached 1.5-fold change **(Table S5b).**

For GNCA, 37 genes had significantly different expression levels in family history positive (n = 16) versus negative (n = 56) cases **(Table S6a)**, but fold changes were all less than 1.5. Significant differentially-expressed genes were also identified for several tumor clinical characteristics, including late (III/IV) versus early (I/II) stage (n = 90 genes), and high (3) versus low (1/2) grade (n = 89 genes) **(Tables S6b and S6c)**. Lymph node metastasis, the strongest clinical characteristic predictive of survival, was also associated with expression levels in 57 genes **(Table S6d)**.

### 6. Gene Expression and Survival

**Figure 5:**
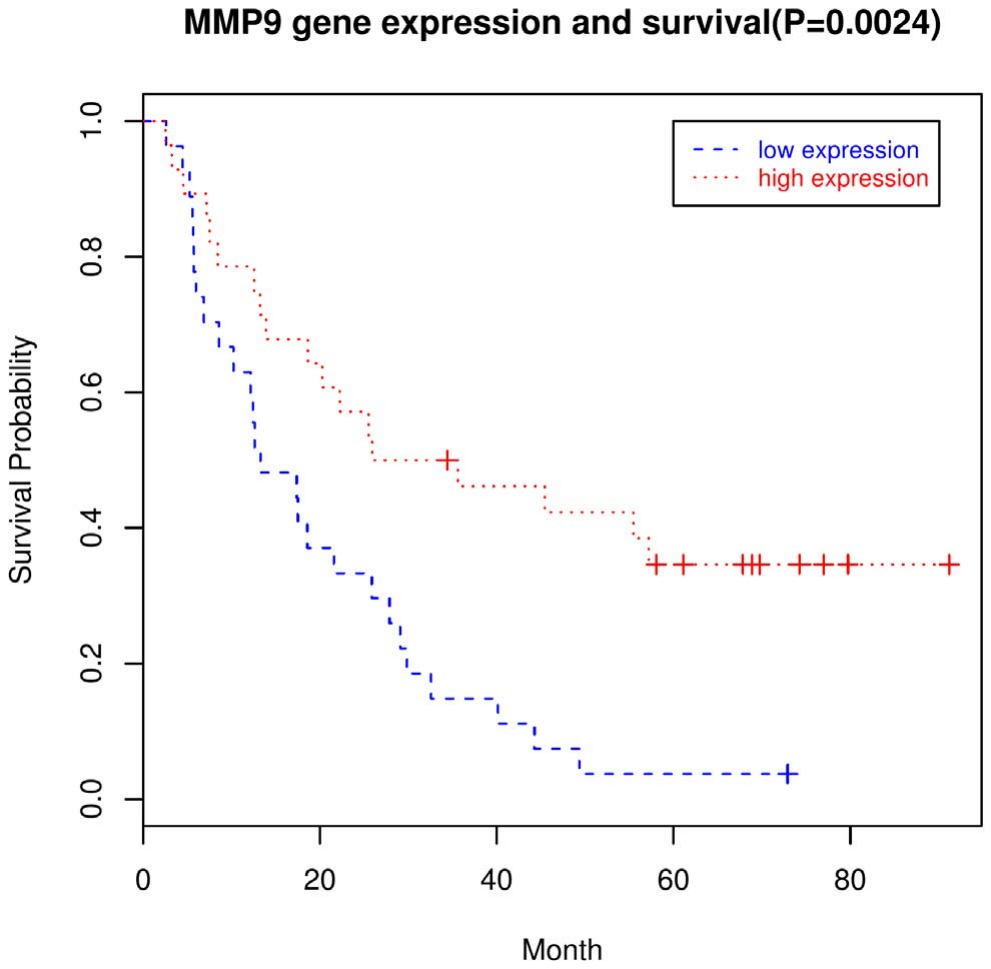
Survival curve for *MMP9* in GCA by Kaplan-Meier analyses. Dotted red lines indicate high (above the median) expression and broken blue lines indicate low (below the median) expression.

**Figure 6:**
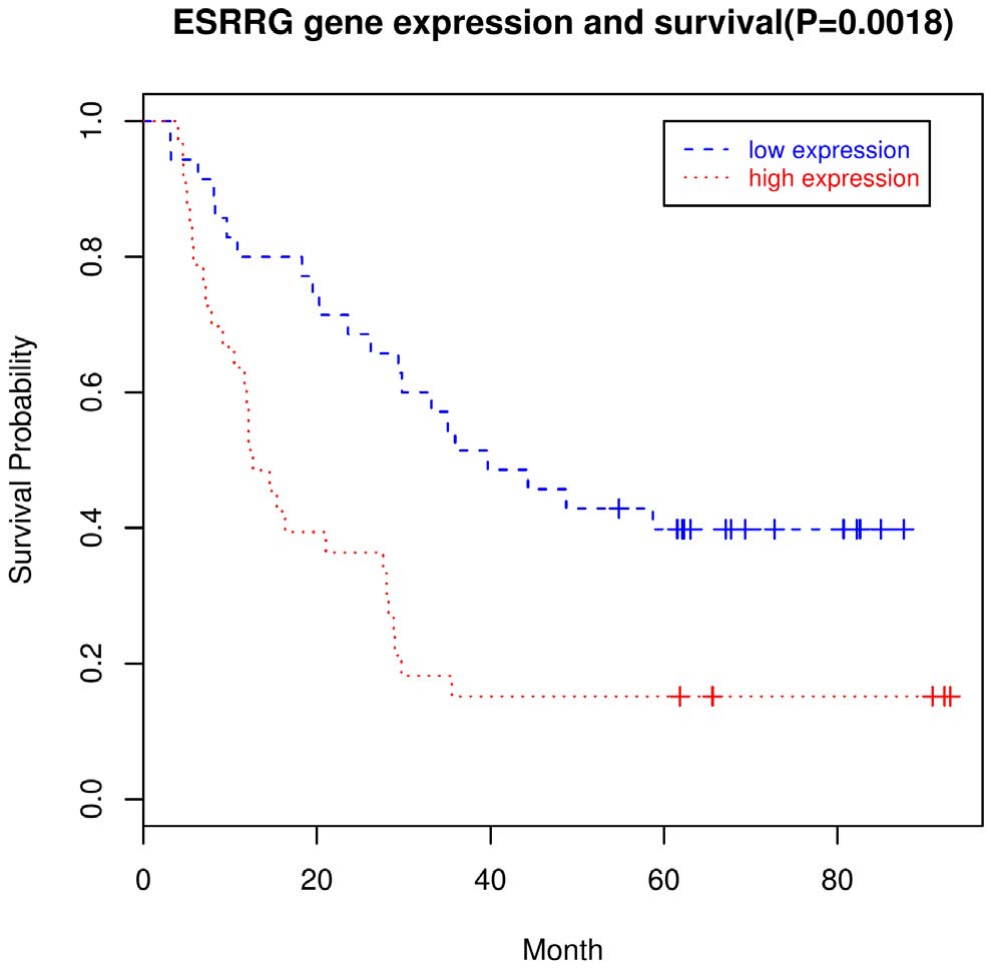
Survival curve for *ESRRG* in GNCA by Kaplan-Meier analyses. Dotted red lines indicate high (above the median) expression and broken blue lines indicate low (below the median) expression.

We evaluated the relation of RNA expression to survival for each of the genes/probesets on the microarray. For GCA, 20 genes were significantly associated with survival (nominal *P*-value <0.05) by log rank tests **(** **Table 2** **)**. An illustrative example of the survival curve for one of these genes (*MMP9)* is shown in **Figure 5**. Eleven genes remained significant after further adjustment for covariates in Cox models. Similar analyses for GNCA showed that 36 genes were significantly associated with survival in log rank tests, including 27 that remained significant after covariate adjustment **(** **Table 3** **)**; a survival curve for one of the 36 (*ESRRG*) is shown in **Figure 6**.

**Table 2:** Genes whose expression was significantly associated with survival in gastric cardia adenocarcinoma cancers (log-rank p-value <0.05; n = 62).

Probeset no.	Probeset	Gene no.	Gene symbol	Cytoband	Fold change	Log-rank p-value	Cox proportional hazard models		
							HR	95% CI	P-value
1	216594_x_at	1	*AKR1C1*	10p15-p14	0.48	0.030	2.00	1.06–3.72	0.033
2	209699_x_at	2	*AKR1C2*	10p15-p14	0.44	0.031	1.60	0.78–3.08	0.210
3	205623_at	3	*ALDH3A1*	17p11.2	0.29	0.037	0.40	0.21–0.77	0.006
4	204290_s_at	4	*ALDH6A1*	14q24.3	0.37	0.031	1.50	0.79–2.92	0.220
5	221589_s_at		*ALDH6A1*	14q24.3	0.29	0.047	1.50	0.79–2.97	0.200
6	217014_s_at	5	*AZGP1/LOC646282*	7q22.1	0.34	0.038	0.59	0.30–1.13	0.110
7	202095_s_at	6	*BIRC5*	17q25	2.02	0.049	1.90	1.01–3.65	0.047
8	205941_s_at	7	*COL10A1*	6q21-q22	3.33	0.008	2.00	1.01–3.83	0.047
9	217428_s_at		*COL10A1*	6q21-q22	2.24	0.008	2.00	1.01–3.83	0.047
10	206212_at	8	*CPA2*	7q32	0.16	0.013	0.52	0.25–1.07	0.074
11	213274_s_at	9	*CTSB*	8p22	2.25	0.004	0.43	0.20–0.91	0.027
12	207912_s_at	10	*DAZ1/DAZ3/DAZ2/DAZ4*	Yq11.223	0.31	0.043	0.54	0.28–1.06	0.072
13	202973_x_at	11	*FAM13A1*	4q22.1	0.47	0.018	2.40	1.16–4.81	0.018
14	207067_s_at	12	*HDC*	15q21-q22	0.49	0.039	0.50	0.26–0.95	0.035
15	210511_s_at	13	*INHBA*	7p15-p13	6.43	0.023	1.40	0.72–2.78	0.320
16	205422_s_at	14	*ITGBL1*	13q33	2.86	0.050	1.60	0.88–3.12	0.120
17	214927_at		*ITGBL1*	13q33	2.17	0.021	1.90	0.90–4.18	0.091
18	209894_at	15	*LEPR*	1p31	0.31	0.037	1.50	0.77–2.95	0.230
19	206334_at	16	*LIPF*	10q23.31	0.02	0.046	0.48	0.25–0.90	0.022
20	203936_s_at	17	*MMP9*	20q11.2-q13.1	2.27	0.002	0.56	0.25–1.23	0.150
21	203675_at	18	*NUCB2*	11p15.1-p14	0.48	0.038	2.20	1.04–4.59	0.039
22	212353_at	19	*SULF1*	8q13.2-q13.3	6.02	0.037	2.30	1.13–4.67	0.022
23	204033_at	20	*TRIP13*	5p15.33	2.13	0.004	2.80	1.44–5.24	0.002

Genes are ordered alphabetically within gastric cancer anatomic subsite.

Abbreviations: HR = hazard ratio, CI = confidence interval.

Hazard ratios are from Cox models adjusted for age, sex, tumor stage, tumor grade, and lymph node metastasis.

**Table 3:** Genes whose expression was significantly associated with survival in gastric noncardia adenocarcinoma cancers (log-rank p-value <0.05; n = 72).

Probeset no.	Probeset	Gene no.	Gene symbol	Cytoband	Fold change	Log-rank p-value	Cox proportional hazard models		
							HR	95% CI	P-value
1	208636_at	1	*ACTN1*	14q22-q24	2.08	0.011	2.40	1.22–4.63	0.011
2	204639_at	2	*ADA*	20q12-q13.11	0.39	0.013	0.41	0.21–0.79	0.008
3	216705_s_at		*ADA*	20q12-q13.11	0.43	0.006	0.40	0.21–0.76	0.005
4	206262_at	3	*ADH1A/ADH1B/ADH1C*	4q21-q23	0.27	0.036	2.50	1.37–4.58	0.003
5	206561_s_at	4	*AKR1B10*	7q33	0.07	0.019	2.50	1.38–4.58	0.003
6	209047_at	5	*AQP1*	7p14	2.15	0.036	2.10	1.12–4.08	0.022
7	207546_at	6	*ATP4B*	13q34	0.01	0.048	2.50	1.34–4.59	0.004
8	209395_at	7	*CHI3L1*	1q32.1	2.31	0.035	0.56	0.30–1.05	0.072
9	209396_s_at		*CHI3L1*	1q32.1	2.18	0.026	0.54	0.29–1.00	0.049
10	202404_s_at	8	*COL1A2*	7q22.1	8.60	0.041	1.70	0.95–3.08	0.075
11	200838_at	9	*CTSB*	8p22	2.62	0.025	0.47	0.26–0.86	0.013
12	204464_s_at	10	*EDNRA*	4q31.23	2.60	0.046	1.80	0.99–3.38	0.053
13	209966_x_at	11	*ESRRG*	1q41	0.13	0.002	2.10	1.11–4.01	0.022
14	221884_at	12	*EVI1*	3q24-q28	0.47	0.044	1.90	1.05–3.59	0.033
15	214752_x_at	13	*FLNA*	Xq28	2.13	0.048	2.10	1.01–4.42	0.046
16	208782_at	14	*FSTL1*	3q13.33	2.42	0.035	2.00	1.06–3.66	0.031
17	218468_s_at	15	*GREM1*	15q13-q15	2.99	0.034	2.00	1.02–3.72	0.043
18	211745_x_at	16	*HBA1*	16p13.3	0.46	0.025	0.56	0.29–1.08	0.082
19	204018_x_at	17	*HBA1/HBA2*	16p13.3	0.47	0.033	0.56	0.30–1.07	0.081
20	209458_x_at		*HBA1/HBA2*	16p13.3	0.46	0.026	0.53	0.28–1.01	0.055
21	211699_x_at		*HBA1/HBA2*	16p13.3	0.45	0.025	0.61	0.32–1.16	0.130
22	217414_x_at		*HBA1/HBA2*	16p13.3	0.46	0.017	0.56	0.30–1.04	0.068
23	209116_x_at	18	*HBB*	11p15.5	0.41	0.032	0.54	0.29–1.00	0.050
24	206858_s_at	19	*HOXC6*	12q13.3	2.35	0.022	2.00	1.04–3.67	0.037
25	208937_s_at	20	*ID1*	20q11	0.48	0.013	0.57	0.31–1.04	0.068
26	202859_x_at	21	*IL8*	4q13-q21	2.23	0.032	0.49	0.26–0.92	0.026
27	219564_at	22	*KCNJ16*	17q23.1-q24.2	0.11	0.045	1.60	0.89–2.90	0.120
28	202202_s_at	23	*LAMA4*	6q21	2.11	0.020	2.20	1.20–4.19	0.011
29	209894_at	24	*LEPR*	1p31	0.41	0.029	2.00	1.07–3.57	0.029
30	218656_s_at	25	*LHFP*	13q12	2.02	0.008	2.30	1.24–4.22	0.008
31	206334_at	26	*LIPF*	10q23.31	0.04	0.029	2.60	1.41–4.76	0.002
32	202291_s_at	27	*MGP*	12p13.1-p12.3	2.10	0.023	2.10	1.11–3.87	0.023
33	210297_s_at	28	*MSMB*	10q11.2	0.27	0.048	2.20	1.20–3.95	0.010
34	212185_x_at	29	*MT2A*	16q13	0.42	0.041	0.57	0.31–1.05	0.072
35	204051_s_at	30	*SFRP4*	7p14.1	4.66	0.037	2.30	1.21–4.34	0.011
36	215223_s_at	31	*SOD2*	6q25.3	2.23	0.014	0.47	0.26–0.86	0.015
37	212667_at	32	*SPARC*	5q31.3-q32	2.84	0.040	1.90	1.00–3.69	0.049
38	218638_s_at	33	*SPON2*	4p16.3	2.08	0.015	2.20	1.22–4.11	0.009
39	214476_at	34	*TFF2*	21q22.3	0.08	0.025	2.70	1.44–4.94	0.002
40	204776_at	35	*THBS4*	5q13	3.25	0.033	1.70	0.92–3.18	0.088
41	208851_s_at	36	*THY1*	11q22.3-q23	2.67	0.028	2.20	1.17–4.14	0.015

Genes are ordered alphabetically within gastric cancer anatomic subsite.

Abbreviations: HR = hazard ratio, CI = confidence interval.

Hazard ratios are from Cox models adjusted for age, sex, tumor stage, tumor grade, and lymph node metastasis.

### 7. Validation of Differentially-Expressed Genes Using Quantitative Real-Time RT-PCR

We performed technical validation experiments for 12 genes in 41 of the 62 GCA cases whose samples still had sufficient RNA quantity after completion of the array study. Four up-regulated (*SULFI, CDC2, TOP2A, BUB1B)* and eight down-regulated (*CA9, CCKBR, PIK3C2G*, *FOS, JUN, KLF4*, *KLK11, NUCB2*) genes were assayed using quantitative real-time RT-PCR (qRT-PCR). Our results showed that gene expression patterns were very similar to RNA array experiment results **(** **Table 4** **and Table S7)**.

**Table 4:** Gastric cardia adenocarcinoma tumor/normal RNA expression fold change for genes validated by RT-PCR and comparison with microarray results (n = 41 cases, 12 genes).

Gene no.	Gene symbol	RT-PCR	RT-PCR expression category (fraction and n)			RT-PCR	Microarray
		fold change(range)	Under-expression(fold change ≤0.50)	Normal expression(fold change 0.51–1.99)	Over-expression(fold change ≥2.0)	fold change(median)	fold change(average)
1	*CA9*	<0.01–0.30	1.00 (41)	0.00 (0)	0.00 (0)	0.01	0.24
2	*CCKBR*	<0.01–0.55	0.98 (40)	0.02 (1)	0.00 (0)	0.22	0.28
3	*KLK11*	<0.01–3.55	0.88 (36)	0.07 (3)	0.05 (2)	0.02	0.31
4	*JUN*	0.03–1.39	0.80 (33)	0.20 (8)	0.00 (0)	0.18	0.35
5	*PIK3C2G*	<0.01–1.15	0.98 (40)	0.02 (1)	0.00 (0)	0.01	0.36
6	*KLF4*	0.02–3.50	0.78 (32)	0.20 (8)	0.03 (1)	0.25	0.40
7	*NUCB2*	0.03–2.58	0.80 (33)	0.15 (6)	0.05 (2)	0.22	0.48
8	*FOS*	0.02–1.70	0.76 (31)	0.24 (10)	0.00 (0)	0.28	0.50
9	*CDC2*	0.08–15.17	0.05 (2)	0.34 (14)	0.61 (25)	2.43	2.39
10	*BUB1B*	0.45–28.71	0.05 (2)	0.32 (13)	0.63 (26)	3.49	2.56
11	*TOP2A*	0.43–50.10	0.05 (2)	0.37 (15)	0.59 (24)	2.90	2.99
12	*SULF1*	0.31–24.42	0.02 (1)	0.20 (8)	0.78 (32)	3.83	4.97

For GNCA, nine differentially-expressed genes (five up- and four down-regulated) were selected for validation by qRT-PCR. Sample pairs for 50 of 72 cases examined on the RNA chip were evaluated for technical replication and showed results similar to those from the array **(** **Table 5** **and Table S8a)**. In addition, matched tumor/normal samples from a new set of 44 GNCA cases were tested for replication validation. Results were consistent with those from the expression array data **(** **Table 5** **and Table S8b)**.

**Table 5:** Gastric noncardia adenocarcinoma tumor/normal RNA expression fold change for genes validated by RT-PCR and comparison with microarray results (n = 94 cases, 9 genes).

Gene no.	Gene symbol	RT-PCR	RT-PCR expression category (fraction and n)			RT-PCR	Microarray
		fold change(range)	Under-expression(fold change ≤0.50)	Normal expression(fold change 0.51–1.99)	Over-expression(fold change ≥2.0)	fold change(median)	fold change(average)
1	*GKN1*	<0.01–1.22	0.96 (90)	0.04 (4)	0.00 (0)	0.00	0.02
2	*KCNE2*	<0.01–1.74	0.96(90)	0.04 (4)	0.00 (0)	0.01	0.09
3	*MSMB*	<0.01–31.05	0.74 (70)	0.13 (12)	0.13 (12)	0.06	0.29
4	*GATA6*	0.02–7.05	0.75 (70)	0.21 (20)	0.04 (4)	0.26	0.36
5	*IGF1*	0.02–76.29	0.16 (15)	0.24 (23)	0.60 (56)	2.40	2.29
6	*FLNA*	0.02–32.90	0.35 (33)	0.21 (20)	0.44 (41)	1.40	2.48
7	*CALD1*	0.10–34.54	0.22 (21)	0.32 (30)	0.46 (43)	1.67	2.49
8	*CKS2*	0.21–27.86	0.08 (7)	0.38 (36)	0.54 (51)	2.05	2.88
9	*DES*	<0.01–50.21	0.34 (32)	0.15 (14)	0.51 (48)	2.01	3.24

Microarray data for 9 genes for GNCA based on 72 cases.

## Discussion

GCA is one of the few malignancies that has increased sharply in developed countries in recent years for reasons that are as yet unexplained, and the molecular events surrounding this gastric cancer remain largely unknown [37], [38]. To better understand the molecular events in gastric cancer and its anatomic subtypes, we profiled gene expression in GCA and GNCA patients from a high-risk population in China using high density RNA expression microarrays. We identified 511 genes whose expression was dysregulated in gastric cancer overall, including nearly one-half (n = 239, 47%) dysregulated in both GCA and GNCA, one-fourth dysregulated in GCA only (n = 128, 25%), and about one-fourth in GNCA only (n = 144, 28%). Associations with family history of UGI cancer hint at genetic susceptibility in etiology, while associations with clinical characteristics and survival suggest potential therapeutic targets for further evaluation.

The common up-regulated genes identified are involved in many pathways related to the development of cancer, including the cell cycle, cellular growth and proliferation, cell cycle checkpoint, extracellular matrix remodeling, and angiogenesis (eg, Wnt signaling and cell cycle checkpoint pathways, such as *SULF1, SFRP4, LEF1, TOP2A,* and *CDC2* [26], [26], [27], [39]–[41], and the integrin signaling pathway (*ARPC1B*, *COL1A1*, *COL4A1*, *FN1*, and *LAMB1))*. Some genes are also related to adaptive immune responses (eg, *CD14*) and tumor metastasis (eg, *CD9*). The common down-regulated genes found in GCA and GNCA are consistent with other studies on gastric cancer using microarrays, such as *AKR1B10*, *ALDH3A1*, *ATP4B*, *CA2*, *IGFBP2*, *KLF4*, *MUC5AC*, *MUC6*, *TFF1*, and *TFF2* [25], [29], [39]. The down-regulated genes in our study are mainly involved in metabolic pathways, digestive system development, or mucosal integrity. Several genes are thought to have specific functions in gastric epithelium, such as *PGC* and *GIF*, implying that dedifferentiation is a common feature of carcinogenesis [26]. *BUB1B* is a spindle-assembly checkpoint gene. A recent report of a case with multiple gastrointestinal neoplasias, including gastric adenocarcinomas, identified a germline homozygous intronic mutation in *BUB1B*, with low levels of *BUB1B* mRNA and protein in lymphocytes and fibroblasts, suggesting that *BUB1B* is a susceptibility gene for this tumor [40]. In our study *BUB1B* was up-regulated in both GCA (2.56 fold) and GNCA (2.11 fold), which is opposite to the case report cited, suggesting that it would be useful to investigate *BUB1B* mutation status in our GCA and GNCA patients.

For genes dysregulated significantly in GCA only, we note a couple of interesting examples here. *SOX9* showed a 2.13-fold change in GCA patients but no significant increase in GNCA cases. *SOX9* (located on 17q24.3-q25.1) is thought to play an essential role in sex determination and marks the precursor cell population during physiological cell replacement including the regenerative process after injury [41], [42]. The expression of *SOX9* has previously been reported in several organs such as pancreas and intestine [41], but not stomach. A recently published study found that “*Sox9* marks a putative adult stem cell population that contributes to the self-renewal and repair of the liver, exocrine pancreas and intestine, three organs of endodermal origin” [41]. *COL2A* is a candidate regulatory target of *SOX9* [42]. In our GCA cases, *COL2A1* was down-regulated (fold change 0.27), which may be a result of *SOX9* up-regulation.

Some of the differentially-expressed genes reported in GCA were also dysregulated in a similar pattern as esophageal squamous cell carcinomas (ESCC) examined from this same high-risk population, such as *CDC25B* and *COL1A2* [43]. This similarity suggests that despite their differences in cell type, GCA and ESCC from this high-risk population of China likely share common genetic and/or environmental factors in their etiology. Evidence for a common genetic influence is evident by results from a recent genome-wide association study which found a shared susceptibility locus in *PLCE1* for both GCA and ESCC [19].

Among the genes significantly dysregulated in GNCA only, *DES* is the only one that showed a different expression directionality in GNCA (3.24 fold change) than GCA (0.85 fold change). *DES (Desmin* on 2q35) encodes desmin, a muscle-specific cytoskeletal protein found in smooth, cardiac, and heart muscles. We identified several genes associated with actin filaments, such as *FLNA*, *ACTN1*, *SVIL* and *TPM1*. Several genes dysregulated only in GNCA were also related with extracellular matrix, such as *EMILIN1* and *TNC*. Studies on *TNC* indicated that up-regulation of *TNC* disrupted cell substrate adhesion [44].

Another purpose of this study was to investigate how gene expression profiles in the tumors differed among patients with different clinical phenotypes. Although we identified a large number of associations at our designated *P*-value threshold of 0.005, only three remained significant after Bonferroni correction for multiple comparisons (ie, *P*<2.36E-06). The most significant associations were for *COL11A1* and *ITGAX* with tumor stage in GNCA, and *UNG* with metastasis, also in GNCA. *COL11A1* [45] and *ITGAX* [46] have both been previously related to tumor stage for other cancers (eg, melanoma, non-small cell lung cancer), but there are no reports for *UNG* and metastasis.

We also sought to evaluate survival by gene expression for genes that were significantly dysregulated. Among the 20 genes related to GCA survival and the 36 genes related to GNCA survival were just three genes that overlapped – *CTSB*, *LEPR*, and *LIPF*. The most significant statistical associations observed with survival (*P*<0.01 in log-rank tests) were for *COL11A1, CTSB*, and *MMP9* for GCA, and *ADA*, *ESRRG*, and *LHFP* for GNCA. Although no studies have yet reported on *COL10A1*, *ADA*, or *LHFP* and cancer survival, *CTSB* [47] and *MMP9* [48] have both been previously associated with survival in gastric cancer, while *ESRRG* expression has been associated with survival in prostate cancer [49].

### Conclusion

This is the first report focused on global gene expression in GCA and GNCA in a high-risk population from Shanxi China. Our study identified hundreds of genes that are changed between tumor and normal tissues as well as genes that distinguish between clinical phenotypes and predict survival. Results described here represent a comprehensive starting point for future efforts to understand etiologic heterogeneity, develop diagnostic biomarkers for early detection, and test molecularly-targeted therapies for gastric cancer.

## Supporting Information

Table S1 Clinical characteristics of cases (**Table S1a**: Clinical characteristics of GCA cases (N = 62); **Table S1b**: Clinical characteristics of GNCA cases (N = 72).
(XLS)

Table S2 Summary of 239 genes that are significantly differentially expressed in both GCA and GNCA.
(XLS)

Table S3 Summary of 128 genes that are significantly differentially expressed in GCA only.
(XLS)

Table S4 Summary of 144 genes that are significantly differentially expressed in GNCA only.
(XLS)

Table S5 Genes significantly associated with patient’s personal/clinical characteristics for GCA (**Table S5a:** Genes related to family history of UGI cancer in GCA cases; **Table S5b:** Genes related to lymph node metastasis in GCA cases).
(XLS)

Table S6 Genes significantly associated with patient’s personal/clinical characteristics for GNCA (**Table S6a:** Genes related to family history of UGI cancer in GNCA cases; **Table S6b:** Genes related to tumor stage in GNCA cases; **Table 6Sc:** Genes related to tumor grade in GNCA cases; **Table S6d:** Genes related to metastasis in GNCA cases).
(XLS)

Table S7 Comparison of gene expression for 12 genes in 41 GCA patients studied using both microarray and qRT-PCR.
(XLS)

Table S8 Comparison of gene expression for 9 genes in 50 GNCA patients studied using both microarray and qRT-PCR (**Table S8a**). Results of gene expression by qRT-PCR for 9 genes in 44 new GNCA cases (**Table S8b**).
(XLS)
